# Experimental Investigation and Simplistic Geochemical Modeling of CO_2_ Mineral Carbonation Using the Mount Tawai Peridotite

**DOI:** 10.3390/molecules21030353

**Published:** 2016-03-16

**Authors:** Omeid Rahmani, James Highfield, Radzuan Junin, Mark Tyrer, Amin Beiranvand Pour

**Affiliations:** 1 Department of Petroleum Engineering, Mahabad Branch, Islamic Azad University, Mahabad 59135-433, Iran; 2 560 Yishun Avenue 6 #08-25 Lilydale, Singapore 768966, Singapore; James03@singnet.com.sg; 3 Department of Petroleum Engineering, FCEE, Universiti Teknologi Malaysia (UTM), Skudai 81310, Johor, Malaysia; radzuan@petroleum.utm.my or r-razuan@utm.my; 4 Universiti Teknologi Malaysia-Malaysia Petroleum Resources Corporation (UTM-MPRC), Institute for Oil and Gas, Universiti Teknologi Malaysia (UTM), Skudai 81310, Johor, Malaysia; 5 Mineral Industry Research Organisation, Wellington House, Starley Way, Birmingham International Park, Solihull, Birmingham B37 7HB, UK; m.tyrer@mtyrer.net; 6 Geoscience and Digital Earth Centre (Geo-DEC), Research Institute for Sustainability and Environment (RISE), Universiti Teknologi Malaysia (UTM), Skudai 81310, Johor, Malaysia; a.beiranvand@utm.my or beiranvand.amin80@gmail.com

**Keywords:** CO_2_ sequestration, forsterite, *ex-situ*, *in situ*, mineral carbonation

## Abstract

In this work, the potential of CO_2_ mineral carbonation of brucite (Mg(OH)_2_) derived from the Mount Tawai peridotite (forsterite based (Mg)_2_SiO_4_) to produce thermodynamically stable magnesium carbonate (MgCO_3_) was evaluated. The effect of three main factors (reaction temperature, particle size, and water vapor) were investigated in a sequence of experiments consisting of aqueous acid leaching, evaporation to dryness of the slurry mass, and then gas-solid carbonation under pressurized CO_2_. The maximum amount of Mg converted to MgCO_3_ is ~99%, which occurred at temperatures between 150 and 175 °C. It was also found that the reduction of particle size range from >200 to <75 µm enhanced the leaching rate significantly. In addition, the results showed the essential role of water vapor in promoting effective carbonation. By increasing water vapor concentration from 5 to 10 vol %, the mineral carbonation rate increased by 30%. This work has also numerically modeled the process by which CO_2_ gas may be sequestered, by reaction with forsterite in the presence of moisture. In both experimental analysis and geochemical modeling, the results showed that the reaction is favored and of high yield; going almost to completion (within about one year) with the bulk of the carbon partitioning into magnesite and that very little remains in solution.

## 1. Introduction

Carbon dioxide (CO_2_) is the principal greenhouse gas released into the atmosphere during fuel combustion, particularly due to the extensive use of fossil fuels for energy production from coal, oil, and natural gas since the industrial revolution [1]. Moreover, atmospheric CO_2_ has recently surpassed 400 ppm, and is predicted to increase to nearly 1000 ppm by the end of the 21st Century [2,3]. The related global temperature rise (exceeding 2 °C) [3] will almost certainly result in irreversible climate change with potentially disastrous consequences.

Researchers have studied ways to mitigate the amount of greenhouse gases released into the atmosphere by sequestration of CO_2_ through different approaches, including aquifer storage, deep sea storage, and mineral carbonation in particular [1,4,5,6,7,8,9,10,11,12,13,14]. Minerals and rocks rich in magnesium (Mg^2+^)/calcium (Ca^2+^) are commonly considered as candidates due to their wide availability, low cost, and environmentally benign nature. [8,12,15] During mineral carbonation, CO_2_ reacts with Mg^2+^ or Ca^2+^-rich minerals (e.g., olivine and gypsum) to form solid carbonates, which are expected to be stable over geologic time periods. The minerals olivine [(Mg,Fe)_2_SiO_4_] and forsterite [Mg_2_SiO_4_], containing up to 33.6 wt. % Mg, have the highest capacity to trap CO_2_ as magnesium carbonates, and a high rate of dissolution among rock-forming silicate minerals [16]. Formation of magnesite from forsterite, the Mg-end member of the olivine solid solution series, is thermodynamically favorable based on the negative Gibbs free energy of Reaction (1).

1/2Mg_2_SiO_4_ + CO_2_→MgCO_3_ + 1/2SiO_2_ + 95 kJ/mol (Rx. 1)

According to Lackner *et al.* [17] the chemical reactions in mineral carbonation process can be very slow under ambient conditions and, therefore, activation processes such as exposure to acid, heat [8], and water [18,19,20,21] have been used to accelerate the carbonation rate. These processes are tedious and require additional energy input, which has made acid/heating approach less attractive than some others. The rates of carbonation can be raised by increasing surface areas of the mineral or its intermediates and by elevating the temperatures, resulting in lower kinetic constraints [15,16,22,23,24,25,26,27,28,29]. Moreover, an *in situ* CO_2_ sequestration system with mineral carbonation can be treated as a fully-coupled problem between rock deformation, pore-fluid flow, heat transfer, mass transport, and chemical reaction processes. Three types of models are commonly employed in computational petroleum geoscience and engineering research methodologies. These approaches are geological/geochemical (conceptual), mathematical, and/or numerical simulation. 

The purpose of this study was to do *ex situ* (laboratory) studies of the factors affecting the rates of (forsterite) mineral carbonation (*i.e.*, particle size, water vapor, and reaction temperature) in support of an *in situ* geochemical conceptual model. A transport reaction modeling software PHREEQC (version 2.18, US Geological Survey (USGS), Reston, WV, USA), was applied for simulating the chemical reactions and transport processes in the forsterite mineral carbonation process. Pre-treatment of forsterite in a quantitative equivalent of mineral acid (HCl), *i.e.*, dissolution to neutrality, was taken as a suitably fast process (in extracting most of the Mg ion) for practical experimentation. One advantage of this process is that the formation of magnesium carbonate (MgCO_3_) releases heat, which can in principle be cycled back to other endothermic steps (see Reactions (2)–(4) in Section 3.3) through heat integration in a commercial process.

## 2. Results and Discussion

### 2.1. Mineral Characterization

The elemental composition of the peridotite mineral was determined by XRF analysis as MgO (51.9%), SiO_2_ (41.1%) as major components with minor levels of FeO, Al_2_O_3_, Na_2_O, K_2_O, and CaO (see details in Table 1).

**Figure 1:**
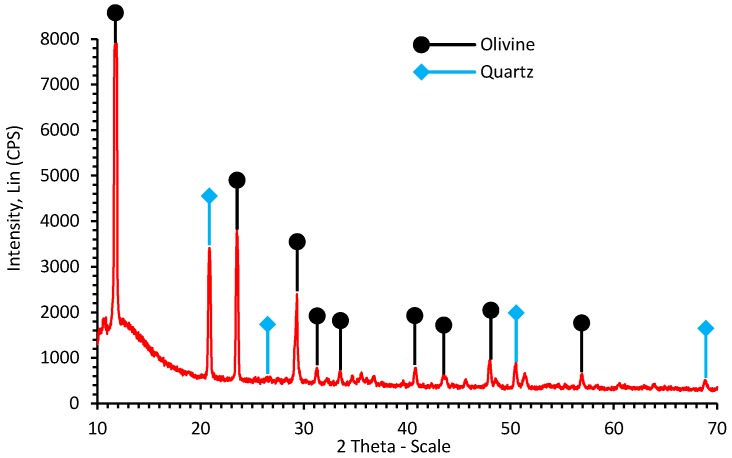
XRD pattern of the starting peridotite mineral.

XRD analysis of the HCl-cleaned starting material (Figure 1) shows a characteristic pattern of olivine (2θ = 11.8°, 23.6°, 29.3°, 31.1°, 33.6°, 40.8°, and 43.6°), with some contamination by quartz or free silica (2θ = 20.9°, 26.5°, 50.5° and 68.7°). In view of the fairly low level of Fe and its own capacity for carbonation (as FeCO_3_), modeling studies (*vide infra*) were based for simplicity on the pure forsterite composition (Mg_2_SiO_4_), the Mg-end member of the olivine solid solution series.

**Figure 2:**
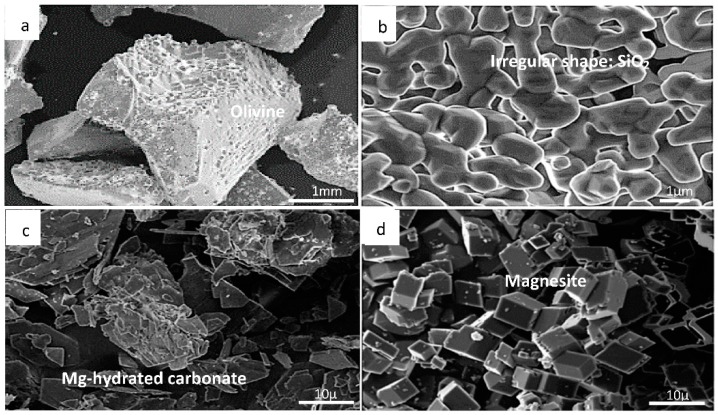
SEM images showing (**a**) the fresh olivine mineral, and morphological changes during chemical pretreatment and carbonation: (**b**) the leached/neutralized sample in the presence of humid CO_2_ at 150 °C during 15 min; (**c**) 90 min; and (**d**) 120 min.

**Figure 3:**
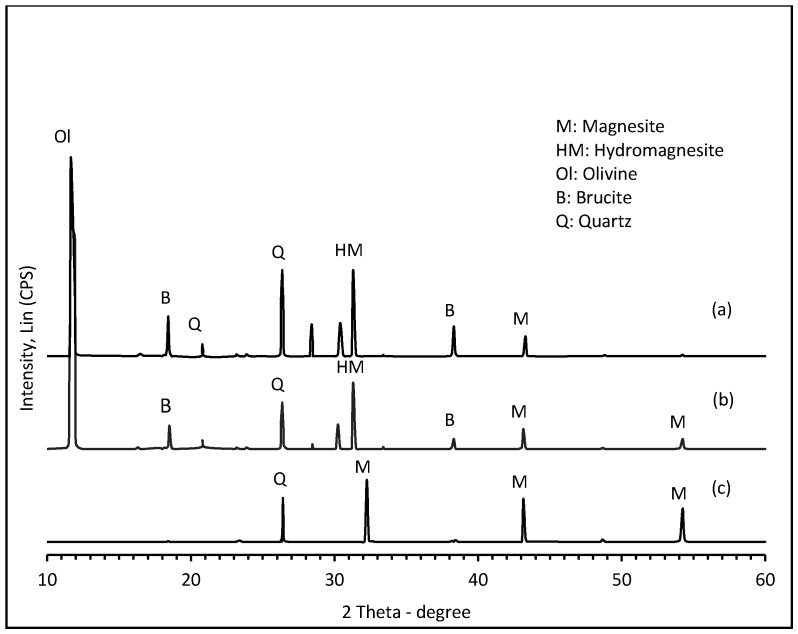
XRD patterns showing structural changes in the olivine mineral during progressive carbonation: (**a**) after acid leaching, neutralization, and initial exposure of the damp residue to humid CO_2_ (P_CO2_ = 4.8 bar, T = 150 °C) during 15 min; (**b**) after 90 min; and (**c**) after 120 min.

Morphological and structural changes in the olivine (nominal composition Mg_1.84_Fe_0.16_SiO_4_) at various stages during chemical pretreatment and carbonation, viz., after leaching in HCl, neutralization and precipitation of Mg^2+^ [as Mg(OH)_2_], and exposure to humid CO_2_ at 4.8 bar and 150 °C, are best seen by SEM micrographs and powder XRD in Figure 2 and Figure 3, respectively.

By SEM, the fresh olivine sample (Figure 2a) consisted typically of polycrystalline grains in the millimeter size range. After acid leaching and neutralization of the sieved fraction <75 μm (Figure 2b), the grains became finer (<5 μm) and the structure more amorphous. In agreement with the findings of Bearat *et al.* [30] and Kwon *et al.* [18], this is likely due to an amorphous SiO_2_ residue after leaching, any (hexagonal) brucite likely being nanocrystalline. Under humid CO_2_, the development of polyhedral and more sheet-like crystallites (Figure 2c) was evident, probably representing (hydrated) magnesium carbonate, and finally (Figure 2d), the rhombohedral habit typical of magnesite (MgCO_3_) was evident.

Figure 3 shows XRD data obtained after the same stages of treatment in parallel with the SEM analyses. Diffractogram 3a reveals that although unreacted olivine (Mg_1.84_Fe_0.16_SiO_4_ main reflection at 2θ = 11.8°) is predominant after only 15 min exposure to humid CO_2_, hydromagnesite (HM = Mg_5_(CO_3_)_4_(OH)_2_**•**4H_2_O main reflection at 2θ = 31.3°) and a little magnesite (M = MgCO_3_ main reflection at 2θ = 43.15°), had already formed from brucite [Mg(OH)_2_] precursor, itself created in the leaching/neutralization stage. At longer exposure times (Figure 3b,c), reflections due to brucite and hydromagnesite were progressively replaced by those of magnesite (2θ = 32.25°, 43.15°, 54.25°). The ultimate disappearance of olivine is intriguing since it implies direct steam-activated carbonation of the (chemically un-pretreated) mineral, for which evidence has been reported elsewhere [31]. The abundance of quartz (as co-product) cannot be taken as a reliable indicator of the progress of carbonation because it is also a phase contaminant in the original mineral (see Figure 1). Furthermore, similar chemical treatment (flux extraction of Mg^2+^) from serpentinites did not produce quartz but instead a silica residue of unusual structure [27]. The unsystematic peak intensity of quartz seen here by XRD may be due merely to local inhomogeneity in the samples.

### 2.2. Effect of Particle Size on the Carbonation of Olivine

**Figure 4:**
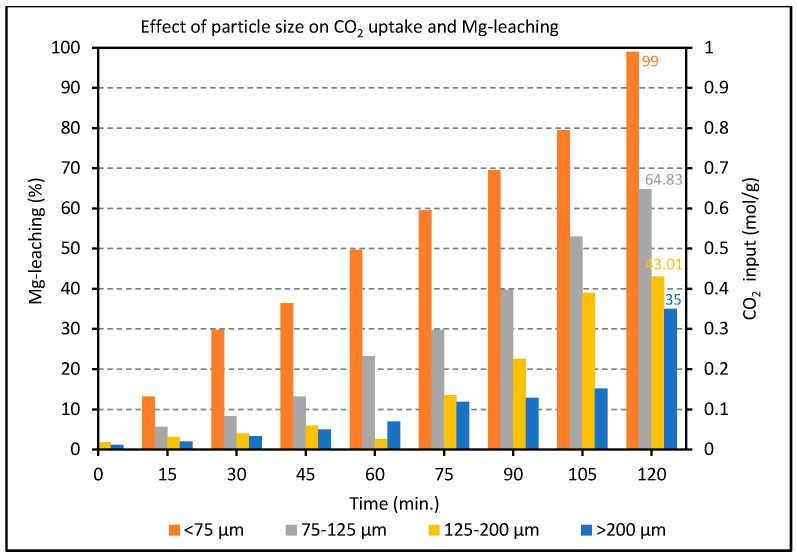
Effect of particle size on Mg^2+^ leaching over a range of time intervals as measured by the volume of CO_2_ uptake in the subsequent mineral carbonation process (at 175 °C and 2 h).

The leaching rate was found to be much slower than the optimal carbonation rate (achievable at 175 °C *vide infra*), such that the production of magnesite in these experiments reflects mainly variations in the rate and efficiency of extraction of soluble Mg^2+^ ion by acid treatment. Figure 4 shows that decreasing the grain size from >200 μm to <75 μm caused the limiting degree of Mg^2+^ leaching to increase from 35% to 99%, respectively. It is well-known and intuitively obvious that the reaction rate and carbonation degree can be raised by increasing the surface area, e.g., by grinding/sieving, as shown for example by Garcia *et al.* [32]. In this work, the smallest particle size (d < 75 μm) is the only one offering the prospect of full leaching (99%) in a practical time. As a rule of thumb, 1–2 h for a terrestrial (*ex-situ*) process to achieve >90% conversion is taken as a realistic practical target to limit the scale (and associated costs) of any future installation for CO_2_ sequestration. This particle cut and leaching procedure (2 h at 60 °C) were therefore applied as standard for all samples in subsequent tests described below.

### 2.3. Effect of Temperature on the Carbonation of Olivine

**Figure 5:**
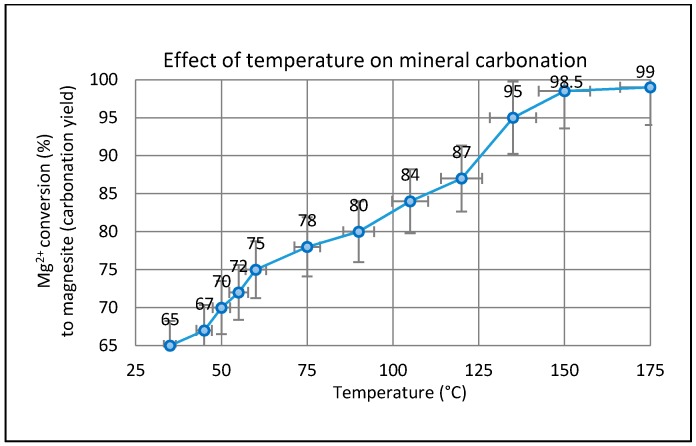
The effect of reaction temperature in gas-solid carbonation of fully extracted Mg^2+^ ion (as Mg(OH)_2_) to yield MgCO_3_ (P_CO2_ = 4.8 bar, humid CO_2_, *t* = 2 h).

The temperature dependence of the carbonation process was studied over the interval from ambient to 175 °C. The effect of temperature on the amount of Mg conversion after standard leaching treatment (99% extraction) is illustrated in Figure 5. As expected, the temperature had an important effect on the mineral carbonation process consisting of Mg extraction and subsequent MgCO_3_ precipitation. The amount of Mg converted at the desired temperatures was measured continuously several times. The maximum extent of carbonation of (99%) was attained at temperatures in excess of 150 °C. The quantification of the MgCO_3_ formed in the mineral carbonation process was carried out by titration against HCl at room temperature. Pokrovsky *et al.* [33] demonstrated that the dissolution rate of magnesite at 150 °C is lower than at 25 °C whereas the rates at 100 and 150 °C in acidic solutions are almost the same [34], suggesting a strong decrease of the apparent activation energy above 100 °C. According to Saldi *et al.* [34] the tendency for dissolution rates of MgCO_3_ to decrease with increasing temperature could benefit CO_2_ sequestration efforts by making magnesite more resistant to dissolution in deeper (hotter) strata, thus preserving the petrophysical integrity of deep carbonate-rich confining reservoirs.

It is clear from Figure 5 that several competing factors in the carbonation process reach equilibrium in the temperature range between 150 and 175 °C. Probably the key factor is the relative humidity (RH). Previous work has shown that carbonation accelerates on cooling below 200 °C in a fixed partial pressure of steam or RH ≥ 20% [35]. Since the presence of liquid water has been shown to be important, it can also be argued that the effect of temperature on the solubility of CO_2_ may influence the carbonation rate. Chen *et al.* [36] indicated that increasing temperature from 25 °C to 150 °C generally helps the precipitation of magnesite. The carbonate solubility product (K_sp_), Henry’s constant (K_H_), and the first- and second-order dissociation constants of carbonic acid (Ka_1_, Ka_2_) are all functions of temperature. Increasing the reaction temperature decreases the value of K_sp_ and increases the K_H_ value, which lowers the amount of CO_2_ gas in solution at a given pressure. The dissociation constants of carbonic acid (Ka_1_ and Ka_2_) are also increased by increasing temperature and promoting solution speciation and carbonation. Thus, increasing temperature has conflicting effects, lowering the level of dissolved CO_2_ gas via its effect on K_H_ but promoting magnesite precipitation via effects on Ka_1_, Ka_2_, and K_sp_. Furthermore, temperature impacts on the kinetics of magnesite precipitation [37] and also affects the type of Mg-carbonate formed. It is well-known that magnesite precipitation kinetics at ambient temperatures are exceedingly slow, and that metastable hydrated carbonates such as hydromagnesite, dypingite, and nesquehonite almost invariably form instead.

### 2.4. Effect of Water Concentration

**Figure 6:**
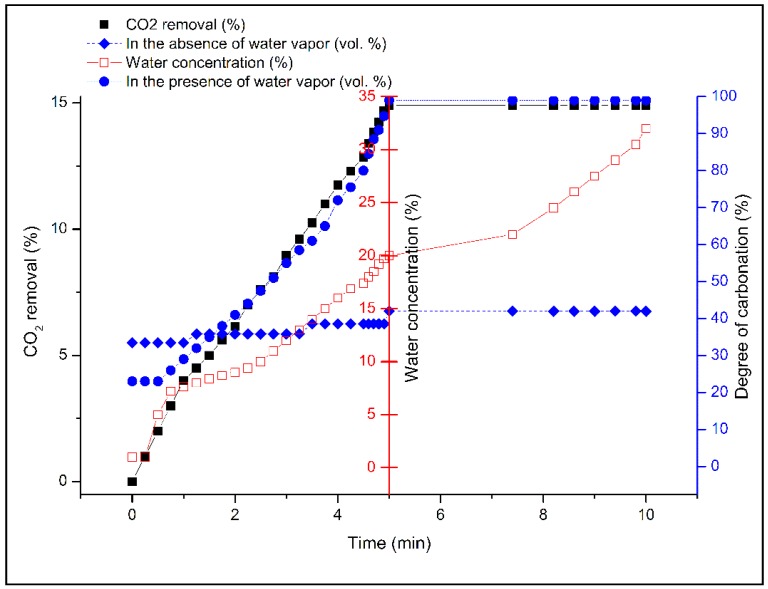
Effect of the presence or absence of water vapor on the rate of CO_2_ absorption at 175 °C on 10 g Mg_2_SiO_4_ (d < 75 μm) activated by chemical pre-treatment and exposed to 0.5 L/min CO_2_ gas (P_CO2_ = 4.8 bar, P_H2O_ = 1.6–6.4 bar or 18%–72% RH).

Formal carbonation of forsterite [Reaction (1)] does not involve water molecules explicitly, so any beneficial effect of added water is clearly a kinetic effect. As shown in Figure 6, an indication of the importance of water is seen in the modest level of CO_2_ removal in its absence. By analogy with recent works on brucite and serpentinites, almost regardless of the CO_2_ pressure utilized, the presence of water vapor in high relative humidity appears crucial to obtaining practical carbonation rates [20,31,38], evidently by establishing a highly polar thin-film aqueous overlayer that facilitates CO_2_ ingress into the bulk particle. This is supported by independent studies simulating *in situ* or geochemical carbon sequestration where CO_2_ was assumed to be in the supercritical state. Felmy *et al.* [39] studied high-surface-area forsterite in the presence of water-saturated *sc*CO_2_. They concluded that the nature of the water in contact with the reacting surface is a key factor in the enhanced magnesite formation. When excess water was added to the forsterite particles, a thin water film was formed on the forsterite surface promoting magnesite formation. Loring *et al.* [40] declared that this water film provides a distinctive situation for the magnesite formation by decreasing the effective Mg^2+^ dehydration energy and simplifying the transformation of nesquehonite to magnesite. Otherwise, the presence of liquid water can allow the formation of magnesium bicarbonate in solution that decomposes upon drying to magnesium carbonate. Moreover, Schaef *et al.* [41,42] revealed that the addition of water to the saturated system noticeably increases the rate of mineral carbonation, facilitating the overall conversion of nesquehonite to magnesite. No evidence of further carbonation was observed under unsaturated conditions below 50 °C. A similar promoting effect of water on *brucite* carbonation under *sc*CO_2_ was reported by Loring *et al.* [43] using *in situ* Fourier-Transform Infrared (FTIR) spectroscopic experiments.

To investigate the effect of humidity, the concentration of water vapor was set at various levels, 5, 10, and 20 vol % prior to carbonation of the brucite extract at 175 °C. As illustrated in Figure 6, above 5 vol % steam, the degree of carbonation increases by almost double in the presence of water vapor such that within 5 min, complete removal of CO_2_ (15 vol *%*) was achieved. However, levels of water vapor exceeding 20 vol % had no additional effect on the rate of removal of CO_2_. It can be concluded that water vapor is able to solvate CO_2_, generate carbonate ions and protons [44], and increase the carbonation degree of Mg(OH)_2_ as derived from olivine. Moreover, the aforementioned modest uptake of CO_2_ (~7 vol *%*) under “dry” conditions may be due to adventitious water not fully removed from the Mg(OH)_2_-containing residue. Vitillo [45] declared that in the presence of water vapor, MgCO_3_ crystalline phase reappeared increasingly, while the magnesium oxide periclase (MgO) phase gradually disappeared. These observations are well in agreement with the thermodynamic data on MgO, Mg(OH)_2_, and MgCO_3_ systems. The promoting effect of water may be attributed to faster reaction kinetics by offering alternative routes to magnesite via hydrocarbonate intermediates such as dypingite.

As a comparison to related work on Mg(OH)_2_ in the literature, Siriwardane and Stevens [46] reported good absorption kinetics and reasonable capacity for CO_2_ in plug-flow reactor experiments over a promoted brucite (~3 mol CO_2_/kg or 20 mol %) in “moist” helium at 200 °C. However, the promotional effect of water *per se* was not explored and the sorbent surface area was low (~2.5 m^2^·g^−1^). This compares with our thermogravimetric work [38] showing that Mg(OH)_2_ extracted from the mineral is typically obtained in high-surface-area form (~25 m^2^·g^−1^). These are evidently important factors in attainment of almost quantitative (~100%) carbonation at lower temperature (150–175 °C) in this work, specifically the higher water levels utilized and the better dispersion of brucite derived from the mineral. Based on the thermodynamic equilibrium of Mg(OH)_2_ formation from MgO, Siriwardane and Stevens [46] showed that is likely to form Mg(OH)_2_ under the high steam environment, which accounts for the subsequent CO_2_ uptake. It is important to note that the Mg(OH)_2_ system has the far lower heat of sorption. This confirms that the regeneration heat (input) needed to displace CO_2_ from MgCO_3_ by water (to form Mg(OH)_2_) is significantly lower than that required for the decomposition of MgCO_3_ (to MgO + CO_2_).

### 2.5. Kinetic Analysis of Mg Extraction by HCl

**Figure 7:**
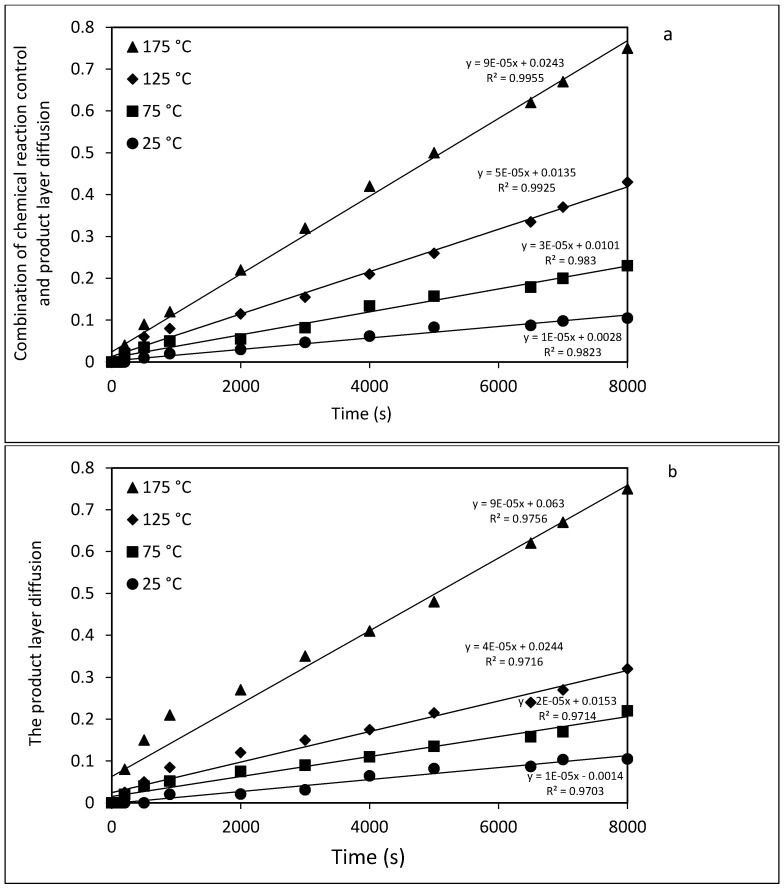
Kinetic analysis of olivine dissolution rate (in HCl) by plotting the combination of chemical reaction control and product layer diffusion (**a**) and the product layer diffusion (**b**) *vs.* time at various reaction temperatures.

Different kinetic analyses including expressions for product layer diffusion, film diffusion, chemical reaction control, and a combination of chemical reaction control (Equations (4)–(7), respectively, in Section 3.5), were used to evaluate the integral rate data. The extent of forsterite dissolution, X_E_, is taken as fitting parameter but this is actually measured from the amount of magnesite, *i.e.*, the extent of carbonation (= R_CO2_ in Equation (2)), because dissolution is much slower than carbonation (of the Mg(OH)_2_ extract). Direct carbonation of unreacted forsterite is probably even slower. Thus, dissolution is rate-determining in the overall process. The two best-fit results are illustrated in Figure 7a,b, but the first, a combination of *chemical reaction control* and *product layer diffusion* (Equation (7)) provided the highest correspondence with the measured data. Thus, it can be concluded that a combination of chemical reaction control and product layer diffusion is rate-limiting for Mg extraction.

**Figure 8:**
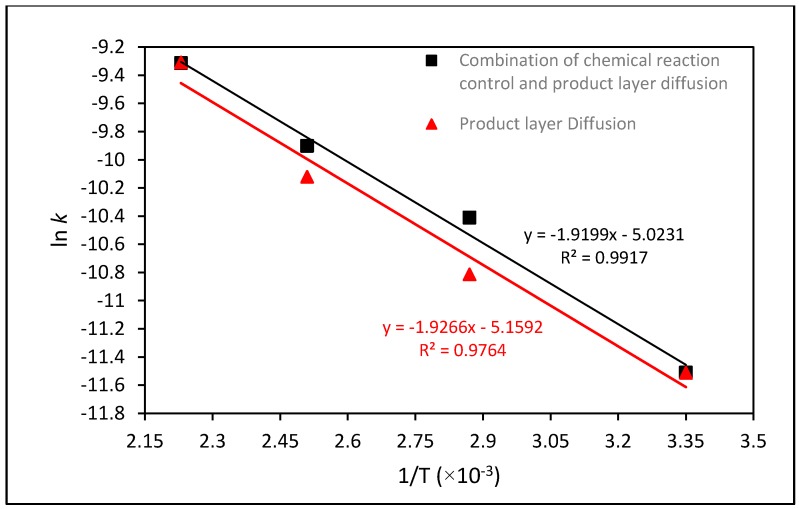
The Arrhenius plots for the extraction of Mg from forsterite using two selected models of the combination of product layer diffusion and chemical reaction control and the product layer diffusion.

Activation energies (*E_a_*) for mineral dissolution were determined from simple log/log plots of the time-independent rate k at various temperatures. These values are presented in Table 2, from which the Arrhenius plots shown in Figure 8 were obtained. Once again, the quality of fit was best for the combination of product layer diffusion and chemical reaction control (R^2^ = 0.9917) as compared to product layer diffusion only (R = 0.9764). Considering these models as the controlling mechanisms during the dissolution of forsterite, the *E_a_* value was 15.5 kJ/mol for product layer diffusion control, and 16.0 kJ/mol for the combination of product layer diffusion and chemical reaction control. It is likely that the chemical reaction is initially rate-limiting but product layer diffusion gradually becomes rate limiting as the product layer of silica builds up and the unreacted surface area decreases. According to Gharabaghi *et al.* [47] a low value of *E_a_* indicates that product layer diffusion is rate-controlling, Therefore, considering the values of multiple regression coefficients for different models and calculated *E_a_* for two selected models, it could be concluded that the dissolution rates of forsterite are kinetically regulated by the combination of chemical reaction control and product layer diffusion.

The rate constants generated in this study on forsterite are faster than corresponding rate constants reported by Pokrovsky and Schott [48], who only worked at the temperature of 25 °C. Comparison of photomicrographs showing the surface of forsterite presented by Pokrovsky and Schott [48] and the samples from this study (see Figure 2) demonstrates they consist of euhedral and larger crystals due to small adhering particles and their agglomeration in acid solution at the temperature of 25 °C. In the present study, the rate constants are increased because of the far higher density of activated sites per unit surface area at higher temperatures.

### 2.6. Thermodynamic Considerations on the Mineral Carbonation

As regards the effect of temperature on the system, two physical mechanisms interact; the increase of olivine solubility with temperature and, conversely, the reduction of magnesite solubility with increasing temperature. Heat treatment was reported by O’Connor *et al.* [49] to remove sorbed water and activate the mineral surface. They suggested that the carbonation phase occurs quickly in the olivine powders at high temperatures, from which we expect an increase in the available reactivity of mineral surface with removal of sorbed water and CO_2_ uptake by the mineral surface and, therefore, more rapid reaction.

Additionally, elevated temperature causes an increase of the olivine dissolution rate, which enhances the overall reaction kinetics. Our results concur with this (see Figure 5) up to the maximum temperature studied (175 °C). The lowest CO_2_ concentration considered was 0.5% (volume fraction of CO_2_ unconsumed) indicating olivine samples have efficiently precipitated CO_2_ as carbonate phase, which is in agreement with the findings of Kwon *et al.* [18]. Interestingly, the CO_2_ sequestration capacity of olivine mineral seen here over the temperature range of 150–175 °C was higher than in previous studies [50,51,52,53,54,55]. We tentatively suggest that this effect is due to either an increase of the available surface area or available moisture (or a combination of both factors). At the highest temperatures, (e.g., the range of 150–175 °C), Hänchen *et al.* [56] suggest the olivine samples released more Mg into the solution, which seems a reasonable assumption to adopt here and is confirmed by the calculation shown below. At lower temperatures (less than 100 °C), King *et al.* [12] found that MgCO_3_ precipitation was kinetically hindered due to the high activation energy for the de-solvation of the strongly hydrated Mg^2+^ ions (Reaction (2)). According to Hänchen *et al.* [57] during low temperature experiments, hydromagnesite [Mg_5_(CO_3_)_4_(OH)_2_•4H_2_O] was precipitated from the solution (Reaction (3)) instead of magnesite (MgCO_3_). When the temperature was increased (up to 125 °C), hydromagnesite was transformed to MgCO_3_ albeit slowly (more than 15 h).

Mg_2_SiO_4 (sol)_ + 2CO_2 (gas)_ + 2H_2_O _(liq.)_→2 MgCO_3 (sol)_ + H_4_SiO_4 (aq.)_ (Rx. 2)

5Mg_2_SiO_4 (sol)_ + 8CO_2 (gas)_ + 20H_2_O _(liq.)_→2[4MgCO_3_•Mg(OH)_2_•4H_2_O] _(sol_) + 5H_4_SiO_4 (aq.)_ (Rx. 3)

The work of Hänchen *et al.* [57] is intriguing, as we found the evidence of hydromagnesite formation in our samples, suggesting that this phase may be as a metastable “intermediate” phase [38] with respect to magnesite, but kinetically favored as a first reaction product under certain conditions (e.g., at low temperature conditions).

### 2.7. Geochemical Modeling

**Figure 9:**
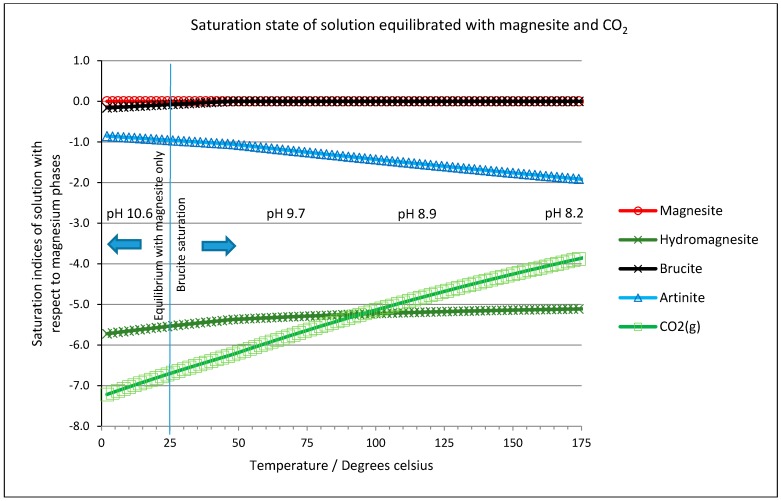
Saturation state of a solution equilibrated with magnesite and carbon dioxide gas (P_CO2_ = 4.8 bar), allowing brucite to precipitate above 25 °C.

Equilibrium simulations were performed using PHREEQC (version 2.18—Parkhurst and Appello [58] with data from the Lawrence Livermore National Laboratory (LLNL, Livermore, CA, USA, supplied with the code). The first, very simple calculations examined the saturation state of a solution (initially pure water) when equilibrated with either magnesite, or both magnesite and carbon dioxide gas. Figure 9 shows the saturation index of both solutions with respect to magnesium phases which could be formed from the elements in the simulated system. In each case, the solutions suggest saturation with brucite, Mg(OH)_2_, would be reached above 25 °C, so the simulations were repeated, but allowing brucite to precipitate when it would otherwise be oversaturated at ambient pressure and it is these conditions which are shown in Figure 9. It is interesting to note that the solution remains undersaturated with respect to hydromagnesite at all temperatures and the influence of CO_2_ saturation is negligible. This reinforces the view that hydromagnesite is an artifact of rapid precipitation, *i.e.*, a process under kinetic control, in preference to magnesite (in line with experiments by Konigsberger *et al.* [59] and Power *et al.* [60]), which, under equilibrium conditions, is the dominant magnesium-bearing phase.

Secondly, attempts were made to simulate the reaction of CO_2_ gas with forsterite in the presence of water, with and without varying amounts of HCl. These results also suggest considerable disequilibrium, in that magnesite would *not* be the dominant “sink” for magnesium in the presence of the silicate ions liberated from forsterite dissolution. Examination of the saturation state of solutions in which CO_2_ was sequentially added to forsterite-water mixtures, always suggested a greater tendency for sepiolite precipitation, in preference to magnesite. Over the temperature interval 1 to 175 °C, CO_2_ gas was sequentially “reacted” (numerically) with an excess of forsterite in the presence of water and these simulations were repeated in the presence of HCl to represent any carry-over of the acid from the previous processing step. Although we recognize that the presence of hydrochloric acid has little influence on the thermodynamic equilibrium position, it has a major role in promoting a rapid increase in the forsterite dissolution kinetics, so it was considered here. In all cases, thermodynamic equilibrium was only reached when sepiolite was allowed to precipitate, which effectively prevented magnesite formation. No evidence of other phases being important in these reactions was seen and the influence of residual HCl was negligible.

**Figure 10:**
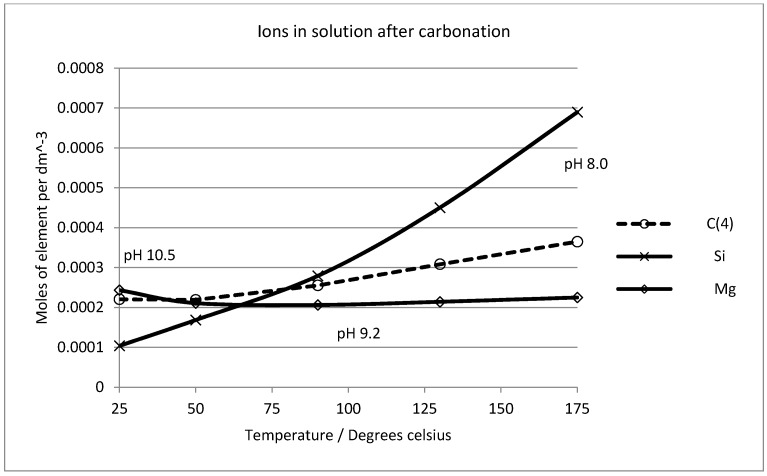
Solution chemistry predicted after reaction of excess forsterite (1 mol) with carbon dioxide gas (0.2 mol of CO_2_) over the temperature interval (25 °C to 175 °C).

Experimentally, no evidence of sepiolite formation was seen and magnesite certainly dominates the solid phase assemblage after carbonation in this work and in the previous studies cited above. Our observations are that forsterite dissolves and magnesite and amorphous silica precipitate as reaction products (see Figure 2 and Figure 3). Experimental studies on the stability of sepiolite have demonstrated that if the silica activity value is high in alkaline solutions, sepiolite precipitates [61,62,63]. From this, we must assume that the kinetics of sepiolite formation are very slow indeed and consequently it was excluded from the mineral assemblage in subsequent calculations. Figure 10 shows the solution chemistry predicted in a system where forsterite is carbonated by gaseous CO_2_ in the presence of water. One mole of forsterite (in excess) was sequentially reacted with 0.2 mol of CO_2_ gas, allowing equilibrium to be reached with both amorphous silica and all possible magnesium phases except sepiolite (which was prevented from forming in the simulations). At all temperatures between 25 °C and 175 °C carbonation is predicted to cause dissolution of forsterite and precipitation of magnesite and amorphous silica, with no other magnesium-bearing phases reaching equilibrium, except sepiolite which was allowed to remain oversaturated in the pore solution.

**Figure 11:**
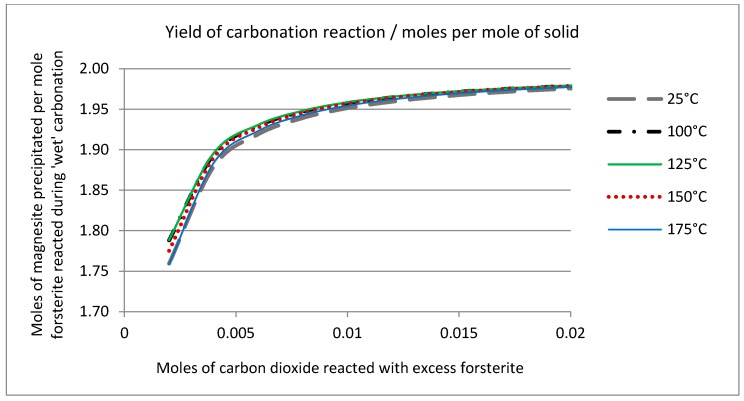
Solid phase chemistry predicted after reaction of excess forsterite (1 mol) with carbon dioxide gas over the temperature interval (25 °C to 175 °C) expressed as moles of magnesite precipitated/moles forsterite reacted, as a function of carbon dioxide consumed.

**Figure 12:**
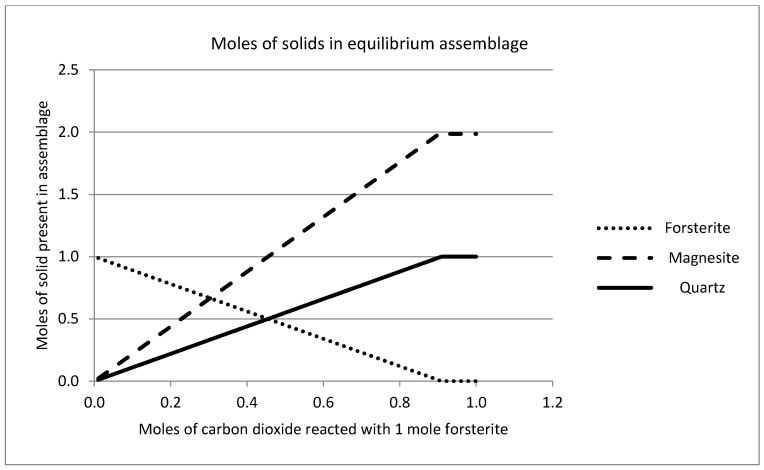
Solid phase chemistry predicted after reaction of one mole of forsterite with excess carbon dioxide gas at 25 °C, showing the stoichiometric relationship between moles of reactant (forsterite) consumed to moles of product (magnesite and quartz) precipitated.

The solid phase chemistry is shown in Figure 11, which plots the ratio of forsterite dissolved against magnesite precipitated (mole ratio) against the number of moles of carbon dioxide consumed by the reaction. This is in effect the “yield” of the reaction, showing the moles of product formed as a function of the moles of reactant consumed. The theoretical stoichiometry (see Equation (1), above) is that two moles of forsterite react with one mole of CO_2_ to produce one mole of magnesite and one mole of amorphous silica. These simulations suggest that this condition is reached quite rapidly. After reaction of only 0.9 mol of CO_2_, the reaction is 97% complete in stoichiometric terms; that is to say the bulk of the CO_2_ reacts to form magnesite and very little partitions into the liquid phase (*cf.* Figure 10). Figure 11 shows that the temperature does have an effect on the extent of reaction and efficiency of carbonation in terms of the overall yield. The pseudo steady-state conditions established after reaction of 0.2 mol of CO_2_ are maintained if further reaction is simulated, such that the solution chemistry remains constant, as does the ratio of forsterite and CO_2_ consumed to the quantities of amorphous silica and magnesite precipitated. This is shown in Figure 12, where an excess of carbon dioxide eventually exhausts the reserve of forsterite, maintaining the stoichiometry of the reaction throughout. It must be stated that since the simplistic geochemical reaction path modeling is used in this study, many physical processes, such as material deformation, pore-fluid flow, heat transfer, advection, diffusion/dispersion, are ignored, although they should be considered in future work, as they are expected to be important during industrial-scale-up. In addition, because the chemical dissolution-front instability is also neglected in this study, many important factors, such as mineral reactive surface area, mineral dissolution ratio, solute dispersion, medium anisotropy, medium and fluid compressibility, have also been ignored. To consider these factors appropriately, more comprehensive chemical-transport modeling will be required.

## 3. Experimental and Modeling Section

### 3.1. Materials

Three kilograms of olivine mineral were excavated and collected from different depths of the Mont Tawai peridotite stratum in Malaysia. Although this source is local, the results can be considered as broadly representative of peridotite as the mineral composition is typical. The samples were crushed by a mechanical grinder and sieved into four different size ranges: <75 µm, 75–125 µm, 125–200 µm and >200 µm. Subsequently, they were dried to a constant weight at 120 °C for 2 h. Ten grams of each size fraction were mixed separately in 500 mL of 0.01 M hydrochloric acid (HCl, QRëC reagent grade, 37%) solution to remove impurities.

The olivine samples were characterized using scanning electron microscopy (SEM, S-4700 Hitachi, Tokyo, Japan) and X-ray diffraction (XRD, X’Pert powder, PANalytical, Almelo, The Netherlands), while elemental composition was measured by X-ray fluorescence (XRF, PW-1410, PANalytical, Almelo, The Netherlands). The carbonation yield of the olivine was determined using a total carbon analyzer (TCA, CS844, LECO Corp., St. Joseph, MI, USA).

### 3.2. Experimental Apparatus

**Figure 13:**
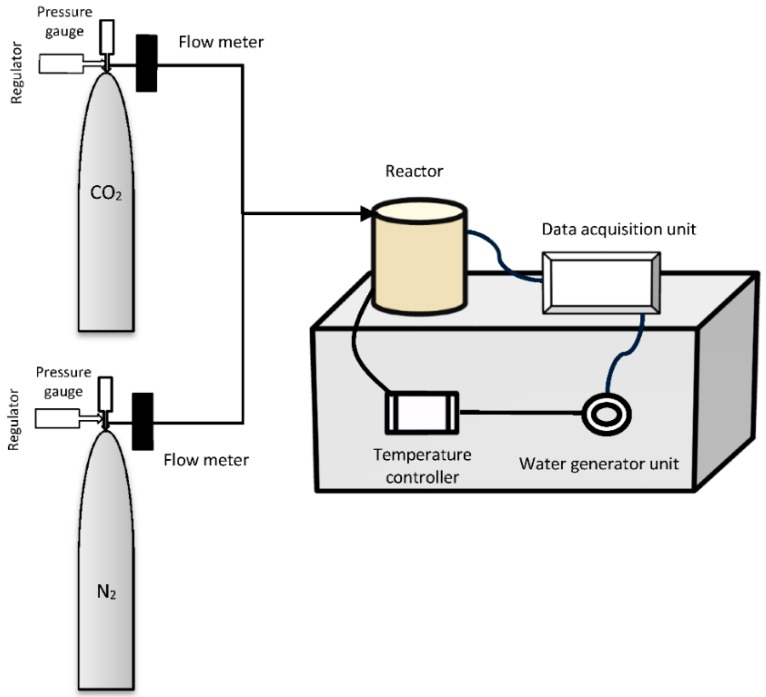
Schematic diagram of experimental set-up for CO_2_ sequestration in chemically pretreated peridotite mineral from Mount Tawai, Malaysia.

The experimental equipment for CO_2_ mineralization consisted of a CO_2_ analyzer system mounted in a flow system connected to a cylindrical (500 mm × 10 mm (d)) autoclave reactor with the means to supply diluted CO_2_ and flushing with pure N_2_ (Figure 13). CO_2_ was introduced into the reactor at different partial pressures (up to 30%). Two flow meters (FM-1050, Matheson Tri-gas, Basking Ridge, NJ, USA) were used to control the flow rate of the inlet gases. The autoclave reactor was loaded with mineral and acid, then placed in a furnace where the temperature was measured and controlled using a thermocouple inserted directly into the reactor.

A water vapor generator was used to humidify the gas stream. CO_2_ consumption was measured as the difference between supply and vent level at fixed flow (with integration over time) using an optical IR-sensor (GMP221, Vaisala Oyj, Helsinki, Finland), according to Equation (1):

(1) $${\text{CO}}_{2}\text{uptake }\left(\frac{mol}{g}\right)=\overset{n}{\underset{i}{\sum }}\frac{{\left({\text{pCO}}_{2\text{ in}}-{\text{pCO}}_{2\text{ out}}\right)}_{i}\times \Delta t\times Q}{R\times T\times M}\;$$

where *p*CO_2 out_ and *p*CO_2 in_ are mean value of *p*CO_2_ (atm) at the inflow and outflow (equilibrium supplyP = 4.8 bar), Δt and Q are time interval (min) and flow rate (L/min), respectively, R and T are gas constant (0.082057 l.atm/mol.K) and temperature (K), respectively, and M is the mass of forsterite (g).

### 3.3. Experimental Procedure

In this study, 1 M HCl was used for dissolution of Mg from the mineral matrix of peridotite and the dissolution experiment was conducted in a separate vessel. When a stoichiometric amount of HCl solution was added to olivine powder in a reaction vessel and stirred with a magnetic stirrer at 60 °C for two h, the entire mass formed a slurry. This slurry was then transferred into the autoclave reactor and neutralized by adding a base (NaOH: Fisher Chemicals reagent grade, with purity 99.999%) until the final pH was increased to above seven. Before heating the reactor, it was purged with nitrogen in order to replace the air inside the reactor, then the reactor was pre-heated to 175 °C for 1 h to dry the slurry and cooled to ambient. CO_2_ gas (SFE grade, with a purity of 99.99% contained in a dip-tube cylinder and purchased from MOX Company, KL, Malaysia) was passed through the dried slurry at a typical flue gas level (15 vol %) or 4.8 bar pressure (P_tot_ = 32 bar) in the absence or presence of water vapor. The temperature of the carbonation process was studied over the interval from ambient to 175 °C.

The reactions involved in the extraction of Mg and carbonation are as follows:

4HCl _(liquid)_ + Mg_2_SiO_4 (solid)_ → 2MgCl_2 (aqueous)_ + SiO_2 (solid)_ + 2H_2_O _(liquid)_ (Rx. 4)

In this reaction scheme (Reaction (4)), forsterite dissolves in HCl, forming soluble MgCl_2_ (Mg^2+^ remains in solution), and leaves behind insoluble SiO_2_. [15] Magnesium hydroxide [Mg(OH)_2_] is precipitated by neutralization with NaOH (Reaction (5)). Then, by passing CO_2_, Mg(OH)_2_ is converted to MgCO_3_ in a gas-solid carbonation process (Reaction (6)).

MgCl_2_ + 2NaOH → Mg(OH)_2_ + 2NaCl (Rx. 5)

Mg(OH)_2_ + CO_2_ → MgCO_3_ + H_2_O (Rx. 6)

The effect of water vapor level on carbonation was studied in the range 5–20 vol % H_2_O, corresponding to a range of relative humidity 18%–72% RH. After completion of the experiment, the samples were collected and filtered using <75 µm pore size Whatman filter papers. The MgCO_3_ formed in the sample was quantified by titration against HCl. For the titration, a certain amount of solid (0.5 g) was weighed in a conical flask. Then, 20 mL of 1 M HCl was added into the flask and was allowed about two hours to react with the MgCO_3_. The excess HCl was then back-titrated using 0.1 M NaOH solution. From the difference in titrant volume, the HCl consumed was calculated from which the content of MgCO_3_ was deduced.

### 3.4. Estimation of Carbonation Yield

The extent of CO_2_ mineral carbonation $Y_{CO_{2}}$ was estimated using the TCA method, which is based on the mineralogy of samples tested and the capacity of carbon sequestrated $\;\left(\frac{1}{R_{CO_{2}}}\right)$ [64]. $R_{CO_{2}}$ is considered as the weight fraction of CO_2_ that can be trapped in a specific amount of mineral. According to Gadikota *et al.* [64] the capacity of CO_2_ sequestration in forsterite (Mg_2_SiO_4_) can be expressed as follows:

(2) $$\frac{W_{CO_{2}}}{W_{fo}}=\left(\frac{1}{R_{CO_{2}}}\right)=\frac{Y_{Mg}}{MW_{Mg}}\times MW_{CO_{2}}$$

where $W_{fo}$ and $W_{CO_{2}}$ are weights of forsterite before its mineral carbonation and CO_2_ sequestrated in the solid phase (*i.e.*, magnesite), respectively. $Y_{Mg}$ is the mass fraction of Mg^2+^ in the forsterite (*i.e.*, 34.55%) that can react with carbon dioxide to form stable magnesite. $MW_{mg}$ and $MW_{CO_{2}}$ are the formula weights of Mg^2+^ (48.61 mol/g in the forsterite) and CO_2_ (44.01 mol/g), respectively, in the carbonated forsterite (2MgCO_3_ & SiO_2_—see (Reaction (1))). Therefore, $Y_{CO_{2}}$ is the amount of carbon dioxide sequestrated (as magnesite) relative to the maximum capacity of CO_2_ sequestration in forsterite $\left(\frac{1}{R_{CO_{2}}}=31.27\%\right)$.

(3) $$Y_{CO_{2}}=R_{CO_{2}}\times \left[\frac{3.67\times {\text{weight fraction of carbon in MgCO}}_{3}}{1-\left(3.67\times {\text{weight fraction of carbon in MgCO}}_{3}\right)}\right]\times 100\text{\%}$$

where 3.67 is the CO_2_/C mass ratio. The carbonation yields of the forsterite and the effect on these of key empirical variables—reaction temperature, time, and particle size, are compared below.

### 3.5. Modeling System and Kinetic Analysis

Thermodynamic calculations were performed with the PHREEQC program software (version 2.18) with data from the LLNL [58]. Forsterite is the Mg-end member of the forsterite-fayalite solid solution series, and is included in the LLNL database. This program was used to estimate the dissolution and carbonation of forsterite samples in order to predict CO_2_ uptake processes and potentials. Moreover, thermodynamic equilibrium constants for the mineral carbonation reactions of forsterite were provided by model databases. In doing so, reaction kinetics were implemented by using a BASIC interpreter. The possibility of implementing reactions kinetic into the code as BASIC statements was also used to predict the reaction progress over time. Consequently, the quality and validity of the model system and the determined rate and equilibrium parameters were verified against the results of carbonation experiments with forsterite samples. The data from a sequence of laboratory efforts were applied for that purpose, which were performed in the aqueous autoclave mini reactor.

The kinetic analysis of the forsterite dissolution rates was determined according to “*standard integral analysis Levenspiel’s method*” [65] in Mg-rich solution using HCl. The results were set into several heterogeneous reaction models represented by integral rate equations and then the multiple regression coefficients (*R*) were calculated. The *shrinking core* model described by Dri *et al.* [66] was applied for the constant size of forsterite particles. Based on this method, reaction rates take place at the outer surface of the unreacted particles, and heterogeneous reactions are controlled by the *product layer diffusion* (Equation (4)), *film diffusion* (Equation (5)) and *chemical reaction* *control* (Equation (6)). In addition, the possibility of having a compound effect of “*chemical reaction control*” and “*product layer diffusion*” was investigated using Equation (7).

(4) $$kt=1-3{\left(1-X_{E}\right)}^{\frac{2}{3}}+2\left(1-X_{E}\right)$$

(5) $$kt=1-3{\left(1-X_{E}\right)}^{\frac{1}{3}}$$

(6) $$kt=X_{E}$$

(7) $$kt=[1-3{\left(1-X_{E}\right)}^{\frac{2}{3}}+2\left(1-X_{E}\right)]+[1-3\left(1-X_{E}{)}^{\frac{1}{3}}\right]$$

In these equations. “*t*” is time (s) and “*k*” (s^−1^) and “$X_{E}$*”* denote the rate constant and the extent of reaction, respectively.

## 4. Conclusions

This work has experimentally and numerically modeled the process by which carbon dioxide gas may be sequestered, *in situ* by reaction with forsterite and/or its extracted intermediate brucite (*ex situ*) in the presence of moisture. In both cases, we have found that the reaction is favored resulting in a high carbonate yield; going almost to completion with the bulk of the carbon partitioning into magnesite and that very little remaining in solution. In the presence of water vapor, the degree of mineral carbonation was increased, we suggest, due to an alternative carbonation pathway providing for faster reaction kinetics [64]. Despite the observations made in other studies, we suggest that hydromagnesite is an intermediate in these carbonation experiments but is converted into magnesite on the (hours) timescale of reaction studied here, although the mechanism is as yet unclear. Hydromagnesite is less desirable as final product as it corresponds to only 80% sequestration (relative to magnesite) on a molar basis. Moreover, we recognize that this system is itself not at thermodynamic equilibrium, but maybe take a very long period and recrystallization as sepiolite (Mg_4_Si_6_O_15_(OH)_2_·6H_2_O) is one possible outcome. However, we have found no evidence that this occurs over the period of one year. The clay mineral sepiolite is known to form in nature from dolomite/silica assemblages in the presence of water, but its formation kinetics are slow and we do not expect its formation to occur spontaneously in an industrial carbonation plant. Thus, we propose that the carbonation of readily available forsterite is a viable route for carbon sequestration.

From the computational perspective, a CO_2_ sequestration system with mineral carbonation can be treated as a fully-coupled problem between rock deformation, pore-fluid flow, heat transfer, mass transport, and chemical reaction processes. The results obtained from this study establish the viability of a geochemical model that can be used in to simulate the dynamic processes involved in CO_2_ sequestration in the Mount Tawai peridotite, Malaysia.

## Figures and Tables

**Table 1:** Chemical composition of fresh peridotite mineral (wt. %) as determined by XRF analysis.

Al_2_O_3_	CaO	FeO	MgO	K_2_O	SiO_2_	Na_2_O	Cr_2_O_3_	Volatiles	
								C + CO_2_	H_2_O
0.204	0.061	5.969	51.921	0.005	41.072	0.083	0.034	<0.352	0.291
**Number of ions on the basis of O**									
Al	Ca	Fe	Na	Mg	K	Si	Na	Cr	-
0.001	0.004	0.161	0.001	1.812	0.004	0.995	0.008	-	-

**Table 2:** The rate constant calculation for every experiment at different temperatures.

Kinetic Analysis	*k*	ln *k*	T (°C)	T (K)	1/T
Combination of chemical reaction control and product layer diffusion	9.0243 × 10^−5^	−9.313	175	448.15	0.00223
	5.0135 × 10^−5^	−9.9007	125	398.15	0.00251
	3.0101 × 10^−5^	−10.4109	75	348.15	0.00287
	1.0028 × 10^−5^	−11.5101	25	298.15	0.00335
Product layer diffusion	9.0635 × 10^−5^	−9.3086	175	448.15	0.00223
	4.0244 × 10^−5^	−10.1205	125	398.15	0.00251
	2.0153 × 10^−5^	−10.8121	75	348.15	0.00287
	1.0014 × 10^−5^	−11.5115	25	298.15	0.00335
