# Correction: Rahmani, O., et al. Experimental Investigation and Simplistic Geochemical Modeling of CO_2_ Mineral Carbonation Using the Mount Tawai Peridotite. *Molecules* 2016, *21*, 353

**DOI:** 10.3390/molecules25081799

**Published:** 2020-04-14

**Authors:** Omeid Rahmani, James Highfield, Radzuan Junin, Mark Tyrer, Amin Beiranvand Pour

**Affiliations:** 1 Department of Petroleum Engineering, Mahabad Branch, Islamic Azad University, Mahabad 59135-433, Iran; 2 Department of Natural Resources Engineering and Management, School of Science and Engineering, University of Kurdistan Hewlêr (UKH), Erbil 44001, Kurdistan Region, Iraq; 3 560 Yishun Avenue 6 #08-25 Lilydale, Singapore 768966, Singapore; james_highfield@ices.a-star.edu.sg; 4 Department of Petroleum Engineering, Universiti Teknologi Malaysia (UTM), Skudai 81310, Johor, Malaysia; radzuan@petroleum.utm.my; 5 Mineral Industry Research Organisation, Wellington House, Starley Way, Birmingham International Park, Solihull, Birmingham B37 7HB, UK; m.tyrer@mtyrer.net; 6 Institute of Oceanography and Environment (INOS), Universiti Malaysia Terengganu (UMT), Kuala Nerus 21030, Terengganu, Malaysia; beiranvand.amin80@gmail.com

After careful consideration, we found that Figure 2 [1] was mistakenly misplaced. Referring to Reviewer #2 comment in Round 1, we should have added the corresponding images of forsterite with ones after the HCl (hydrochloric acid) attack. Although we had responded to the comment accordingly, we forgot to insert the new images that could cover this point. The authors would like to apologize for any inconvenience caused to the readers by this change. Replacing this figure will not affect the results or conclusions of the paper. The manuscript will be updated, and the original will remain online on the article webpage, with reference to this correction.

## Figures and Tables

**Figure 2:**
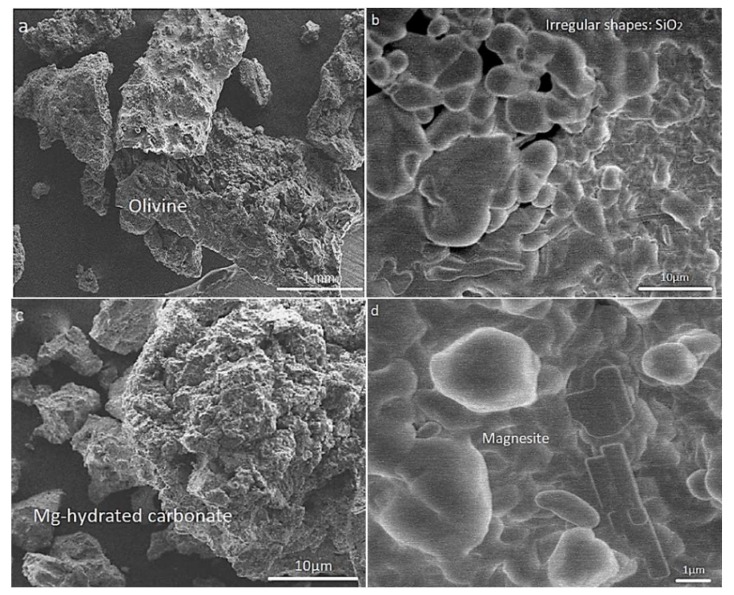
SEM images showing (**a**) the fresh olivine mineral, and morphological changes during chemical pretreatment and carbonation: (**b**) the leached/neutralized sample in the presence of humid CO_2_ at 150 °C during 15 min; (**c**) 90 min; and (**d**) 120 min.

## References

[1] Rahmani O., Highfield J., Junin R., Tyrer M., Pour A.B. (2016). Experimental Investigation and Simplistic Geochemical Modeling of CO_2_ Mineral Carbonation Using the Mount Tawai Peridotite. Molecules.

